# Results of the Italian RESILIEN-T Pilot Study: A Mobile Health Tool to Support Older People with Mild Cognitive Impairment

**DOI:** 10.3390/jcm12196129

**Published:** 2023-09-22

**Authors:** Roberta Bevilacqua, Elisa Felici, Giacomo Cucchieri, Giulio Amabili, Arianna Margaritini, Claudia Franceschetti, Ilaria Barboni, Susy Paolini, Patrizia Civerchia, Alessandra Raccichini, Simona Castellani, Lucia Paciaroni, Giuseppe Pelliccioni, Elvira Maranesi, Lorena Rossi

**Affiliations:** 1 Scientific Direction, IRCCS INRCA, 60124 Ancona, Italy; r.bevilacqua@inrca.it (R.B.); e.felici@inrca.it (E.F.); g.cucchieri@inrca.it (G.C.); g.amabili@inrca.it (G.A.); a.margaritini2@inrca.it (A.M.); c.franceschetti@inrca.it (C.F.); l.rossi@inrca.it (L.R.); 2 Clinical Unit of Physical Rehabilitation, IRCCS INRCA, 60127 Ancona, Italy; i.barboni@inrca.it; 3 Unit of Neurology, IRCCS INRCA, 60127 Ancona, Italy; s.paolini@inrca.it (S.P.); p.civerchia@inrca.it (P.C.); a.raccichini@inrca.it (A.R.); s.castellani@inrca.it (S.C.); l.paciaroni@inrca.it (L.P.); g.pelliccioni@inrca.it (G.P.)

**Keywords:** older people, mild cognitive impairment, memory support, quality of life, resilience, AAL technologies, usability

## Abstract

(1) Background: The RESILIEN-T system addresses the need for innovative solutions to support self-management in older people with Mild Cognitive Impairment (MCI). Despite the increasing prevalence of dementia and MCI, there is a lack of tailored solutions for these individuals. The RESILIEN-T system aims to empower and engage people with cognitive decline by providing a modular platform for self-management and coaching services. (2) Methods: Italian data collected for the RESILIEN-T project involved 62 older participants randomly assigned to the intervention or control group. Data were collected through questionnaires and user interactions with the system over a three-month period. (3) Results: Quantitative outcomes showed no significant differences between the intervention and control groups, except for an improvement in perceived memory capability in the intervention group. The usability assessment indicated a high level of acceptance of the RESILIEN-T system. (4) Discussions: Although no significant improvements were observed in most quantitative measures, the high user engagement and acceptance suggest the potential effectiveness of the RESILIEN-T system. Future improvements could involve integrating smart objects and interactive virtual agents. Overall, RESILIEN-T represents a promising step toward empowering individuals with cognitive impairment in their self-management and decision-making processes.

## 1. Introduction

Worldwide, it is estimated that there is one new case of dementia every three seconds [1]. There are approximately 50 million people living with dementia, and this number is predicted to reach 131.5 million in 2050. Mild Cognitive Impairment (MCI) is another age-related condition in which an individual has mild but measurable changes in cognitive abilities that are greater than would be expected for their age. These changes are noticeable to the affected person and to family members and friends but do not affect the individual’s ability to carry out everyday activities. Approximately 15 percent to 20 percent of people aged 65 or older have MCI. People with MCI, especially MCI involving memory problems, are more likely to develop Alzheimer’s disease or other dementias than people without MCI [2,3]. The cost of cognitive impairment depends on the care setting (community vs. residential) and can fluctuate from an annual cost of EUR 17,300 for a patient with mild dementia cared for in the community (reaching EUR 24,200 and EUR 34,000 for moderate and severe dementia, respectively) [4] to EUR 37,200 for a patient with dementia, at any stage of the disease, placed in a nursing home. 

There is good evidence that a healthy lifestyle and cognitive stimulation can reduce both the risk of developing dementia and its progression [5,6]. As we wait for disease-modifying therapies for dementia, there is an urgency for new strategies to appraise the preferences, feelings and expectations of people with a diagnosis and to leverage and maximize their residual skills.

Despite the large amount of research on how to exploit Information and Communication Technology (ICT) to support the self-management of chronic diseases, there is a lack of clear direction when it comes to self-management by older People with Cognitive Impairment (PwCI). This is due to a persisting bias against considering people with a diagnosis of cognitive impairment as capable of participating in the care sector. This bias has limited the development of ICT solutions to support PwCI and empower them to participate directly in making decisions about their lives. Self-monitoring and self-management could complement traditional clinical approaches by educating PwCI on how to live with the best possible quality of life [7]. In this scenario, ICT solutions could represent an effective tool to encourage PwCI to actively participate in the care decision-making process, allow them to interact directly with health care providers, express their personal health concerns and take initiatives to improve their own health [8].

In this paper, the RESILIEN-T system, developed as part of the AAL project (AAL-2018-5-82-CP), is presented along with the results gathered during a three-month experiment in Italy.

The overall aims of RESILIEN-T are to deploy in the market an innovative modular ICT solution for the self-management of cognitive impairment and to reinforce the self-monitoring ability of people with a diagnosis with the aim of slowing the progression of the disease. In particular, the specific objectives identified by the project are to develop an open, modular and adaptable platform to provide self-management and coaching services to PwCI, integrating informal and professional caregivers as necessary, to be used either autonomously or in conjunction with on-the-market systems for lifestyle monitoring or wearable devices. Moreover, evidence-based applications (apps) to support self-management by PwCI, covering the areas of nutrition, physical social and cognitive activities were developed.

The RESILIEN-T system consists of a modular, integrated and open platform offering different services, according to the needs and expectations of the users. The system provides the PwCI and the informal caregiver with a mobile App to deliver coaching services related to nutrition, physical activity, cognitive exercises and social relationships. The App interacts with a remote, cloud-based platform designed to collect all data generated from the user’s interaction with the App (on the on hand), but which also acts as a repository/feeder for the content delivered to the user through the App, like cognitive training games, information about caring or suggestions on nutrition and physical activity. This solution leverages the most up-to-date scientific evidence to empower PwCI to live an active and meaningful life, maintain independence in daily activities and live safely at home with dignity and satisfaction during the course of their illness. The RESILIEN-T solution covers multiple domains of preventive measures: nutritional guidance, physical exercise promotion, cognitive practice, social activity and positive care planning.

Furthermore, the RESILIEN-T solution responds to the fundamental desire of PwCI to remain living in their own home as opposed to other housing solutions [9] by providing a cheap, easy-to-use and effective ICT solution that will support self-management to help PwCI live an active and meaningful life, maintain independence in daily activities and live safely in their own home without (or with low) cost to the public health system.

## 2. Materials and Methods

This study represents Italian data collected for the RESILIEN-T project. A mixed-methods approach, including quantitative and qualitative assessment tools, was used to collect data on the impact of RESILIEN-T. The study was conceived as a pilot study. The sample size indicated is in line with guidelines for pilot and feasibility studies [9], which are aimed at providing preliminary insight into outcomes, study design and other relevant aspects of a study before conducting a randomized controlled trial. The CONSORT checklist has been added in Supplementary Materials. The study was approved by the Ethics Committee of the Istituto Nazionale Ricovero e Cura per Anziani (IRCCS INRCA) on 26 November 2020.

### 2.1. Subjects

End users were mainly recruited from the Neurology Unit and the Alzheimer’s Evaluation Unit (Memory Clinic) of the IRCCS INRCA, where a team of psychologists identified possible participants who met the following inclusion criteria: older than 65 years with worse memory problems compared to 5 years ago (score of 25 or more on the MAC-Q test). The enrolment phase favored direct contact between professionals and participants, allowing for the preselection of the possible end users to be included on the basis of their clinical and cognitive histories. During the first evaluation session, information on clinical history and cognitive status was collected in line with the protocol. Each participant was contacted via telephone by the researcher and invited to the IRCCS INRCA hospital where the RESILIEN-T project was explained. Elderly persons who agreed to participate were asked to sign the informed consent form. At each stage, the elderly were given the opportunity to refuse participation. Following the study design, 64 participants were recruited and randomly assigned to the RESILIEN-T intervention condition or a control condition for 3 months. A randomization technique based on a single sequence of random assignments was used. A list of random numbers generated using a computer was used, and the subjects were assigned a number based on their order of inclusion in the study. The intervention group was properly trained in the use of the system, and the users went home with a tablet and were asked to start using the system immediately. Users were contacted by telephone according to the scheme agreed upon and shared with all partners and were informed that they could contact the principal investigators at any time. The first delivery took place in January 2022 and the last tablet delivered was in May 2022. All participants used the tablet for at least three months. The participants in the control group were met with twice: the first time at the beginning and the second time after three months. The first control group participants started in February 2022, and the last participants started in May 2022.

A number of informal caregivers were present during the recruitment. However, although they were all in favor of the study and agreed to support the elderly persons during testing, none were willing to participate as an informal participant. The main reasons given by the informal caregivers were that they did not want to interfere with the elderly person’s autonomy, were too busy or did not interact often enough with elderly persons. This aspect meant that no questionnaires were completed by informal caregivers.

### 2.2. Clinical Assessment

A standard assessment was performed for all patients, including clinical history; measurement of cognition with the Montreal Cognitive Assessment (MoCA) [10]; measurement of quality of life with the EuroQoL (EQ-5D5L) [11] and with the Quality of Life in Older Adults with Cognitive Impairment (QoL-AD) [12]; measurement of perceived memory capability with the Memory Assessment Clinics-Questionnaire (MAC-Q) [13]; measurement of usability with the System Usability Scale (SUS) [14]; measurement of the personality with the Big Five Inventory-10 (BFI-10) [15]; and measurement of psychological wellbeing with the Warwick-Edinburgh Mental Wellbeing Scale (WEMWBS) [16]. On the qualitative side, a semi-structured interview on usability, acceptability and satisfaction with the system was conducted. For the purpose of the paper, the qualitative assessment was not included. 

### 2.3. Intervention

**Figure 1:**
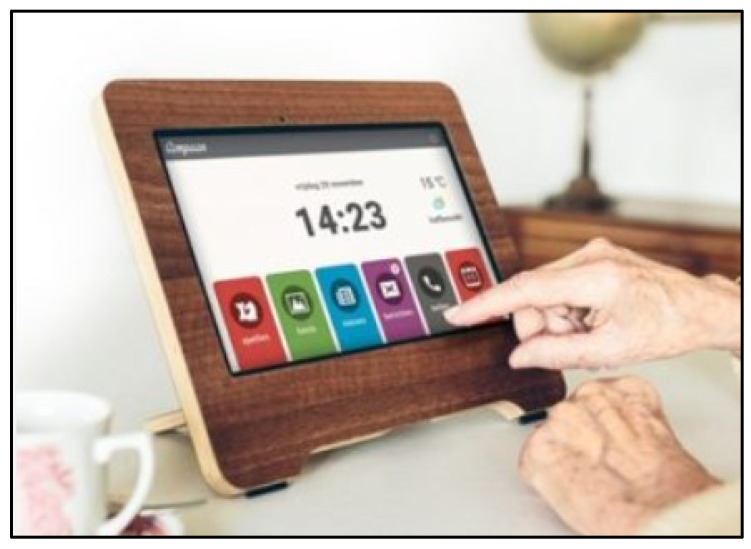
The tablet Compann.

The RESILIEN-T system, designed on the tablet Compann (Figure 1), aims to promote self-management for people with cognitive decline with consequent impacts also for their informal caregivers on social isolation and quality of life. 

Through the tablet’s interface, persons with cognitive decline and their caregivers (if any) received their own accounts to access coaching and keep track of information related to nutrition, physical activity, cognitive exercises and social relationships (Table 1).

The system interacts with a remote, cloud-based platform designed to collect data from each user’s interaction with the system but that also serves as a repository for the contents on cognitive training, nutrition and physical activity on a daily basis. Study participants access RESILIEN-T using the Compann tablet developed and tested for older people. Caregivers can access the system through a freely downloadable App on their smartphone or a dedicated webpage, which has services to interact with their family members through the RESILIEN-T tablet, send messages and/or photos, and make video calls.

Users are also provided with a wearable device (Wave model from iHealth) that operates as an activity tracker, as well as a home monitoring kit, to collect relevant data on daily activities in the domestic environment.

**Figure 2:**
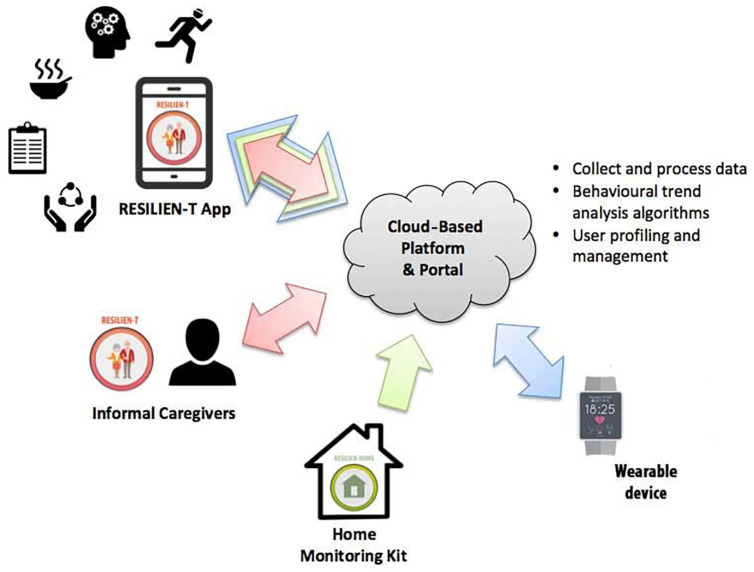
RESILIEN-T ecosystem.

The architecture of RESILIEN-T was designed as an open system so that it will be possible to integrate additional sensors in the future, the use of which was not envisaged in this trial (Figure 2).

Moreover, the project included the completion of two daily questions in the morning and in the evening to assess adherence to the intervention. In the morning, it was asked ‘How do you perceive your health today?’, while in the evening it was asked ‘How much do you appreciate the activities suggested through the RESILIEN-T daily messages?’. The answers were given through a specific scoring system: 1 stands for ‘good’, 2 stands for ‘could do better’ and 3 stands for ‘bad’. The data collected were related to the answers to these questions.

People with MCI are often not included in the research in the technological field while public and patient involvement, especially in an interdisciplinary design and healthcare context, is important to bring together, adapt and develop acceptable and usable technologies. For this reason, during the first stage of the RESILIEN-T project, co-design sessions were conducted to cocreate a common knowledge base among designers, users and other stakeholders regarding goals, needs, weaknesses and expectations to be translated into design and technical requirements for the system’s development. To arrive at the final configuration, the design process of the RESILIEN-T system was divided into three fundamental phases: (a) user research and analysis—to identify users’ daily habits and their lifestyle needs and preferences (i.e., nutrition, physical activity and social relationships); (b) generative and explorative phase—for collecting ideas and merging them into a few scenarios and design concepts; (c) evaluation and assessment stage in co-design workshop—the results are submitted, discussed and improved upon together with the users. The system was conceived to be used by the older end users autonomously in order to enhance the intrinsic capacity of older people.

### 2.4. Statistical Analysis

The descriptive data are presented as the mean and Standard Deviation (SD) for continuous variables or numbers (percentage) for categorical ones. Since we had a small sample size, determining the distribution of the variables was important for choosing the most appropriate statistical method. In line with this, a Shapiro–Wilk test was performed and did not show evidence of non-normality. Based on this, we decided to use a parametric test. In addition, the mean and standard deviation are used to summarize the variables reported. A Pearson’s chi-squared test for categorical variables and Student’s *t*-test for continuous variables were applied to test for statistically significant differences (*p* < 0.05) between CG and TG parameter mean values. Before/after comparisons were assessed with the matched-pairs Student’s *t*-test. A statistical analysis was performed using IBM SPSS Statistics 27.0 software.

## 3. Results

### 3.1. Sample

**Figure 3:**
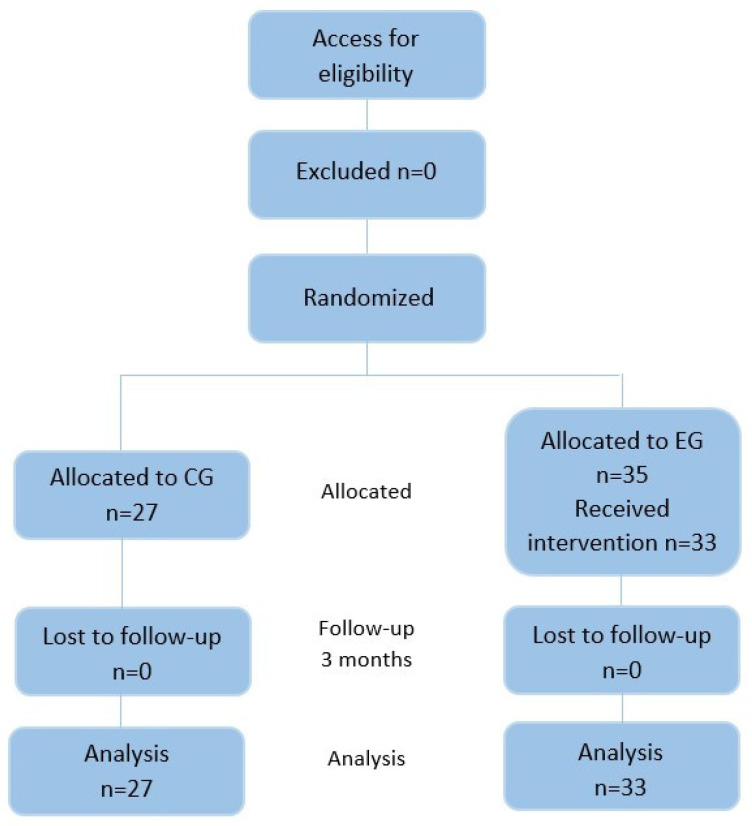
The CONSORT flowchart.

The sample was composed of 62 older participants, divided into two groups: one intervention and one control. In the intervention group, there were 35 people, but two of them withdrew because they lacked interest in the tablet. In the control group, there were 27 people. In the flowchart (Figure 3), the CONSORT (Consolidated Standards of Reporting Trials) is reported.

All participants were retired, and they worked in different areas as employees, engineers, nurses, teachers or were housewives. At baseline, no differences in the demographic characteristics (gender, age and educational level) or in the technological use and competence were found between the two groups, emphasizing the homogeneity of the two groups, as can be observed in Table 2. In particular, both groups were not very confident in using technology, as the test and control groups scored 5.8 and 5.9 on a scale from 1 to 10, respectively. 

Moreover, at baseline, no differences in the evaluation scales (Montreal Cognitive Assessment (MoCA), EuroQoL (EQ-5D5L), Quality of Life in Older Adults with Cognitive Impairment (QoL-AD), Memory Assessment Clinics-Questionnaire (MAC-Q) and Warwick-Edinburgh Mental Wellbeing Scale (WEMWBS) were found between the two groups, emphasizing the homogeneity of the two groups.

### 3.2. Quantitative Outcomes

In order to assess the perceived health status, quality of life and memory status, five standardized quantitative questionnaires were asked of both groups at the beginning and at the end of experimentation. In particular, in Table 3 the following is reported:

− The comparison between T1 and T2 for the Control Group (CG);
− The comparison between T1 and T2 for the Technological Group (TG);
− The comparison between CG and TG at the end of the trial (T2).

Only one significant difference between groups in the results appears. In fact, with regard to the subjective assessment of memory, the MAC-Q shows a slight increase in the technological group and a slight decrease in the control group, with a significant difference between the two groups. A score of 25 or higher, as it resulted in T2 for the technological group, means that the subject reported relevant memory complaints. On the contrary, the MoCA scores showed a nonsignificant decrease in the technological group, but no significant difference was found when compared to the control group. In general, except for perceived memory status, there were no significant differences between groups. The general quality of life was in between the fair and the good perception. The same can be stated for wellbeing, which was assessed using the standard WEMWBS instrument, whose scores ranged from 7 to 35, with higher scores indicating greater positive mental wellbeing. 

### 3.3. Analysis of System Usability

For evaluating the usability of the platform quantitatively, the System Usability Scales (SUS) was used. The 33 older participants successfully completed the System Usability Scales (SUS), a quantitative questionnaire asked to assess the usability of RESILIEN-T. The average score of SUS was 79.1 in which 100 is the maximum and 68 is considered the threshold for a usable system (Table 4) [17]. 

### 3.4. Adherence to the Intervention

For the RESILIEN-T pilot study, the adherence rate was collected through the log automatically recorded by the system and calculated as the number of goals attained as self-reported by the participants, number of steps performed during the day and the daily self-assessment of wellbeing, as collected at two separate moments during the day. The calculation was based on the participants who completed the intervention (this excludes dropouts) [18].

The data from the morning and evening question are reported in Table 5.

Over time, the answers to the morning question did not show any improvement, since the average score in the third month showed a slightly higher value than that in the first month. In the answers for the evening question, there was a slight worsening from the first to the second month, while from the second to the third month, the value remained constant.

The data related to the evening question were also analyzed in groups by goal (Table 6).

A slight improvement in the average answer can already be seen from the first to the second month for both the nutrition and social contacts goals. On the other hand, the physical activity goal was not associated with an improvement in the average score, which presented a slight increase.

**Figure 4:**
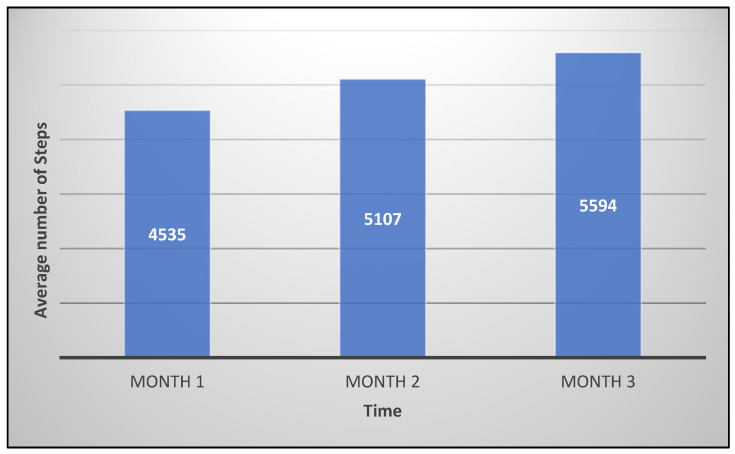
The results for the steps analysis.

Figure 4 shows the number of steps performed by the users during the experiment.

The results outline an improvement in the users’ status given by the increase in the average number of steps over time. It can, therefore, be seen that appropriately incentivized and stimulated users tend to be more inclined to physical and motor activity.

## 4. Discussion

The field trial provided the opportunity to understand the behavior of the participants in relation to an innovative system, the RESILIEN-T, for staying healthy and following personalized goals. The major strength of the study was the really high positive predisposition of the participants to take part in the test due to their interest in having an active role in their own health while also having fun.

A good degree of usability and acceptance of the system has already been retrieved thanks to the cooperation of the users with the research teams. The capability of the system to be adaptive to the changing needs of the user is an essential feature of any assistive technology designed for a population with cognitive decline. Over time, answers to the morning questionnaires did not show any improvement, since the average score in the third month showed a slightly higher value than that in the first month: older people, especially, prefer a system only to help them when it is really necessary and that can be adapted if their needs are changing.

In our study, nonrelevant obstacles were found in terms of usability and acceptability in supporting lifestyle management. From the analysis of the interaction with the system, in fact, it can be observed that the RESILIEN-T system was able to attract users throughout the experiment, assuring sustained adherence to the intervention delivered by the platform. The users, in fact, answered all daily messages sent by the system—i.e., morning and evening messages—and the goal-oriented advice, as shown by the frequency of usage that remained constant throughout the three months. As an indirect benefit of the continuous use of the system, the empowerment of health and digital competencies represents an undeniable achievement to be analyzed in depth in future studies; an intervention based on an e-health system, such as RESILIEN-T, can facilitate patients in managing their own conditions and healthy citizen can benefit from prevention measures, but they are often designed without considering a necessary phase of training before the introduction of the technology. This aspect needs to be included as a complement of the training with the platform and/or as a module of the intervention itself. Nowadays, there is much evidence of the need of an integrated intervention, such as RESILIEN-T, that is able to improve lifestyles, increase adherence to treatments and achieve positive health outcomes, thus supporting patients’ resilience.

Because of the heterogeneity of the aging population, characterized by different levels of intrinsic capacity, disability and motivation, technology for personalized health coaching may represent an effective way to encourage self-management, described as a behavioral intervention to promote goals and reduce health risks also integrated with technology. Despite this, the assessment of the impact of such solutions is still debated in terms of the variables and the duration of the intervention. In the case of RESILIEN-T, we included quality of life as a secondary outcome. What it was observed is that the scores of the QOL-AD and the WEMWBS were improved even if not statistically significant. Despite this result, however, the users did not change their positive attitude toward the system, suggesting the need for further investigation and the adoption of a different study design and outcomes to better understand the impact of RESILIEN-T on the overall quality of life. Moreover, one reason behind this result may lie in the short duration of the trial that did not allow for the detection of a change in a complex dimension such as quality of life. To achieve a better result in the future, it is suggested to perform longer trials, at least 6 months, as well as follow-up. On the services side, future improvements in the system should consider the opportunity of being adaptable to smart objects, such as small social robots, to provide games oriented to memory support using stimuli of emotional salience, for example, autobiographical memories. It will be possible to hypothesize an improvement in the quality of life, not only stability. By adding a more interactive virtual agent, in fact, it may be possible to build empathy and feelings of trust in the e-coach, promoting a solid and effective change in the quality of life.

Even if the quality of life measurements did not reveal a clear improvement, a statistically significant positive evaluation was retrieved in the impact on the cognitive status. In particular, the data show a significant change in the self-perception of memory. In fact, it appears that the group that received the system after 3 months felt an improvement in memory capability. This result represents promising evidence of the effectiveness of the RESILIEN-T system in providing support for the cognitive status of older people with cognitive decline. First, it can be hypothesized that the RESILIEN-T system is capable of addressing specific cognitive functions by offering several activities that provide general stimulation in thinking, concentration and memory skills. Moreover, the RESILIEN-T intervention aimed at training older adults to adopt healthy behaviors allows for an enhancement in cognitive function and enables independence in older adults.

On the technological side, starting from the pilot data, technical developers sought to make the application more usable and customizable to allow participants to set and achieve their goals. The new version of the application used for this field trial allowed participants to choose specific subgoals within the four domains to better customize the notifications they received according to their needs and goals. Participants also received feedback based on their progress and use of the application. In addition, an archive section was added, where participants had the possibility to review the messages divided by the objective and date as often as they wanted. Within the 3 months, no technical problems were detected and the fact that we periodically telephoned the users provided us with an up-to-date picture of their experience and acceptance.

We acknowledge that this study has a number of limitations that should be considered in light of the results. Firstly, the study was conceived as a pilot; thus, the relatively small sample size may have affected our capacity to detect some relationships between an improvement in cognitive status and the use of the system. Secondly, the lack of a long-term follow-up assessment after training must be taken into consideration; evaluating the long-term impact of this type of training will be addressed in future studies. Finally, it is suggested to include the MoCA during the recruitment phase to assess the inclusion/exclusion criteria on the basis of the cognitive status and to evaluate the impact of the intervention in different cognitive domains. In our case, the MoCA was used before and after the intervention, allowing us to control the cognitive status of the participants and the impact of the use of the system. However, in the case of limited resources setting, it is highly recommended to add the MoCA in the recruitment phase. Given the limitations and strength points reported, this pilot study may serve as a first insight into the preliminary data of a more extensive randomized controlled trial.

## Figures and Tables

**Table 1:** App description.

Domain	Objective	Type of Information
Nutrition	Reduce consumption of salt, carbohydrates, sugar and packaged products. Take in the right amount of water, protein, vitamins, fruit and vegetables.	Text 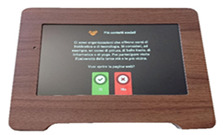 Imagines/video/quiz 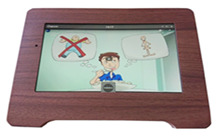 Website 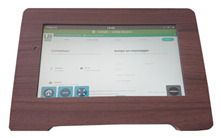
Physical Activity	Balance exercises, regular exercise, stretching, aerobics and walking.	
Cognitive Exercises	Brain-training activities, memory, singing, having a hobby, breaking the routine, language training and riddles.	
Social Relationships	Maintaining social contact, writing, telephoning, video chatting, rules for good conversation, volunteering, social work, group activities and courses.	

**Table 2:** Baseline demographics, general health status and use of technology.

	CG (n = 27)	TG (n = 35)
Gender, n (%)		
Female	15 (55.6%)	17 (48.6%)
Male	12 (44.4%)	18 (51.4%)
Age, mean ± SD	74.2 ± 8.1	75.8 ± 8.1
Educational level, n (%)		
Primary education	13 (48.2%)	18 (51.4%)
Secondary education	12 (44.4%)	12 (34.3%)
University or higher	2 (7.4%)	5 (14.3%)
General health status, n (%)		
Fair	7 (25.9%)	8 (22.9%)
Good	17 (63.0%)	23 (65.7%)
Excellent	3 (11.1%)	4 (11.4%)
Comfortable with technology, mean	5.9	5.8
Use of technology for, n (%)		
Tracking diet	1 (3.7%)	2 (5.7%)
Tracking physical activity	7 (25.9%)	8 (22.9%)
Finding local events	16 (59.3%)	23 (65.7%)
Navigation	11 (40.7%)	19 (54.3%)
Communication	27 (100%)	33 (94.3%)
News	-	26 (74.3%)
Appointments	8 (29.6%)	-

CG = Control Group; TG = Technological Group; SD = Standard Deviation.

**Table 3:** Differences in the T1 vs. T2 indicators for the cases and controls.

Scale	TG			CG			CG vs. TG
	T1	T2	*p*	T1	T2	*p*	*p*
EQ5D	77.1 ± 11.9	79.3 ± 13.5	0.390	73.3 ± 13.0	75.7 ± 12.1	0.079	0.931
WEMWBS	24.7 ± 4.8	24.9 ± 4.1	0.761	24.5 ± 4.3	25.2 ± 4.1	0.098	0.545
QoLAD	31.6 ± 6.6	32.2 ± 5.8	0.489	34.6 ± 6.2	33.8 ± 6.2	0.264	0.241
MACQ	26.5 ± 2.3	25.7 ± 2.2	0.006	25.9 ± 2.4	26.2 ± 2.5	0.152	0.003 *
MoCA30	24.8 ± 2.3	25.1 ± 2.5	0.237	25.3 ± 2.1	25.1 ± 1.9	0.381	0.164

CG = Control Group; TG = Technological Group; WEMWBS = The Warwick-Edinburgh Mental Wellbeing Scale; QoLAD = Quality of Life in Alzheimer’s Disease; MACQ = Memory Assessment Clinics-Questionnaire; MoCA30 = 30 items the Montreal Cognitive Assessment; * *p*-values for the Student’s *t*-test.

**Table 4:** Detail and summary of the SUS scores.

Item	Score, Mean ± SD
SUS1—I think that I would like to use this system frequently	3.77 ± 0.84
SUS2—I found the system unnecessarily complex	1.34 ± 0.68
SUS3—I thought the system was easy to use	4.23 ± 0.69
SUS4—I think that I would need the support of a technical person	2.11 ± 1.08
SUS5—I found the various functions well integrated	3.80 ± 0.63
SUS6—I thought there was too much inconsistency	1.60 ± 0.65
SUS7—I would imagine that most people would learn quickly Widowed	3.94 ± 0.48
SUS8—I found the system very cumbersome	1.23 ± 0.49
SUS9—I felt very confident using the system	3.97 ± 0.62
SUS10—I needed to learn a lot of things before I could get going	1.77 ± 0.88
SUS Score	79.1 ± 12.6

SD = Standard Deviation.

**Table 5:** The results of the morning and evening questions.

	Month 1 (Mean ± SD)	Month 2 (Mean ± SD)	Month 3 (Mean ± SD)
Wellness score (morning question)	1.47 ± 0.33	1.59 ± 0.47	1.60 ± 0.51
Feedback score (evening question)	2.08 ± 0.29	2.09 ± 0.33	2.09 ± 0.37

SD = Standard Deviation.

**Table 6:** Mean value and standard deviation of the feedback scores for each goal for each month.

	Month 1 (Mean ± SD)	Month 2 (Mean ± SD)	Month 3 (Mean ± SD)
Cognitive activity goal	2.15 ± 0.35	2.17 ± 0.38	2.12 ± 0.46
Physical activity goal	2.08 ± 0.42	2.18 ± 0.43	2.18 ± 0.36
Social contacts goal	2.15 ± 0.47	2.08 ± 0.41	2.12 ± 0.42
Nutrition goal	2.04 ± 0.30	2.00 ± 0.38	2.02 ± 0.50

SD = Standard Deviation.

## Data Availability

The datasets generated, used and analyzed during the trial and its preceding pilot trial are or will be available from the corresponding author upon reasonable request.

## Supplementary Materials

The following supporting information can be downloaded at: https://www.mdpi.com/article/10.3390/jcm12196129/s1.
